# A Novel Ex Vivo Approach Based on Proteomics and Biomarkers to Evaluate the Effects of Chrysene, MEHP, and PBDE-47 on Loggerhead Sea Turtles (*Caretta caretta*)

**DOI:** 10.3390/ijerph19074369

**Published:** 2022-04-05

**Authors:** Laura Bianchi, Silvia Casini, Lorenza Vantaggiato, Agata Di Noi, Alfonso Carleo, Enxhi Shaba, Alessandro Armini, Francesco Bellucci, Giovanni Furii, Luca Bini, Ilaria Caliani

**Affiliations:** 1 Functional Proteomics Laboratory, Department of Life Sciences, University of Siena, Via A. Moro, 2, 53100 Siena, Italy; laura.bianchi@unisi.it (L.B.); lorenz.vantaggiato@student.unisi.it (L.V.); enxhi.shaba@unisi.it (E.S.); luca.bini@unisi.it (L.B.); 2 Department of Physical, Earth and Environmental Sciences, University of Siena, Via Mattioli, 4, 53100 Siena, Italy; francescbelluc@gmail.com (F.B.); caliani4@unisi.it (I.C.); 3 Department of Life Sciences, University of Siena, Via P. Mattioli, 4, 53100 Siena, Italy; agata.dinoi@student.unisi.it; 4 Department of Pulmonology, Hannover Medical School, Carl-Neuberg-Straße 1, 30625 Hannover, Germany; carleo.alfonso@mh-hannover.de; 5 Department of Biotechnology, Chemistry and Pharmacy, University of Siena, Via A. Moro, 2, 53100 Siena, Italy; alessandro.armini@unisi.it; 6 Centro Recupero Tartarughe Marine Legambiente, Molo di Ponente, 71043 Manfredonia, Italy; furii@virgilio.it

**Keywords:** skin, whole blood, proteomics, PAHs, phthalates, biological effects, marine reptiles

## Abstract

The principal aim of the present study was to develop and apply novel ex vivo tests as an alternative to cell cultures able to evaluate the possible effects of emerging and legacy contaminants in *Caretta caretta*. To this end, we performed ex vivo experiments on non-invasively collected whole-blood and skin-biopsy slices treated with chrysene, MEHP, or PBDE-47. Blood samples were tested by oxidative stress (TAS), immune system (respiratory burst, lysozyme, and complement system), and genotoxicity (ENA assay) biomarkers, and genotoxic and immune system effects were observed. Skin slices were analyzed by applying a 2D-PAGE/MS proteomic approach, and specific contaminant signatures were delineated on the skin proteomic profile. These reflect biochemical effects induced by each treatment and allowed to identify glutathione S-transferase P, peptidyl-prolyl cis-trans isomerase A, mimecan, and protein S100-A6 as potential biomarkers of the health-threatening impact the texted toxicants have on *C. caretta*. Obtained results confirm the suitability of the ex vivo system and indicate the potential risk the loggerhead sea turtle is undergoing in the natural environment. In conclusion, this work proved the relevance that the applied ex vivo models may have in testing the toxicity of other compounds and mixtures and in biomarker discovery.

## 1. Introduction

The loggerhead sea turtle *Caretta caretta* (Linnaeus, 1758) is subjected worldwide, and particularly in the Mediterranean Sea, to multiple stresses originating from anthropogenic activities. Fishing, intensive tourism, ship traffic, contaminants, and litter present in the marine environment, together with various pathologies that can affect this species, endanger the wellness and survival of the loggerhead sea turtle. The hazard deriving from contamination is also linked to *C. caretta* longevity and to the possible accumulation in its tissues of persistent contaminants. For these reasons, *C. caretta* is monitored by the IUCN (International Union for Conservation of Nature) and included, since 1996, in the Red List of endangered species.

Among the contaminants that particularly constitute a threat there are legacy compounds, such as polycyclic aromatic hydrocarbons (PAHs), organochlorines (OCs), and heavy metals, but also contaminants of emerging interest, such as phthalates, polybrominated diphenyl ethers (PBDEs), and alkylphenols, which also derive their origin from the consistent presence of plastics in the sea. *C. caretta* is constantly in contact with plastics and often ingests them even in large quantities [1,2,3]. To make things worse, plastics act both as a carrier of contaminants already present in the sea and as a release matrix by leaching [4,5,6,7].

Several studies have investigated the presence of both legacy and emerging contaminants in dead stranded loggerhead sea turtles [8,9,10,11]. On the contrary, there is still little information about alive specimens and about the sub-lethal effects these contaminants may have. In recent years, non-destructive methods of sampling free or hospitalized specimens have been developed focusing on blood, skin biopsies, and excreta as target tissues. A multi-biomarker approach [12] was also settled to monitor the sub-lethal effects of contaminants on *C. caretta*, including genotoxic effects [13] and effects on the immune system [14].

Despite this, a great effort is still needed to study the effects of contaminants in this species and its susceptibility to chemical insult not only on natural organisms but also by using in vitro methodologies that can replace laboratory tests on living animals. In vivo testing is in fact an approach that is generally undesirable from an ethical point of view but also legally inadmissible in the case of protected species. In vitro tests are used more and more frequently in human and mammalian toxicology [15,16,17,18] and can allow us to acquire information representative of toxicant effects at the organism level. However, they have some limitations, such as not being able to fully reproduce the biological processes that occur in the whole organism.

To date, few studies have been published that apply in vitro methodologies to *C. caretta* and other sea turtles, mostly *Chelonia mydas* [19,20,21,22]. They are essentially based on the development of cell lines from various tissues, on which some organic and more often inorganic contaminants, e.g. trace elements, have been tested. These studies investigated toxicological endpoints, such as cytotoxicity, oxidative stress, genotoxicity, and expression of genes [23,24,25,26,27,28,29]. Nonetheless, toxicological effects of many compounds, especially emerging ones, and their mixtures remain unexplored. In confirmation of this, few papers focused on the effects of PAHs on sea turtles, although they have been found in different tissues of these marine reptiles from various areas [12,30,31,32,33,34,35,36] and a positive correlation was reported between PAHs and comet assay [36]. Similarly, PBDE-47 effects in *C. caretta* have not been particularly investigated. However, PBDE-47 was described as accumulated in specimens of the loggerhead sea turtle from the Mediterranean Sea [10] and from the South Carolina and Florida coast [37], and its transfer from the mother to the eggs, with probable development and hatchling size effects, as observed in *C. mydas* [38], was also described [38,39]. Phthalates, easily found in the marine environment, are detected in different tissues of *C. caretta* and *Dermochelys coriacea* [11] and can be transferred also to the eggs [40]. Despite this evidence, toxicological effects of phthalates have not been really investigated in *C. caretta*.

In such a context, the aim of the present study was to develop and apply ex vivo tests, not previously attempted in the loggerhead sea turtle, as an alternative to cell cultures to evaluate the possible effects of emerging and legacy contaminants on *C. caretta*. Notably, ex vivo tissue cultures allow to preserve, at least to an extent, tissue-ultrastructure and biochemical functions, thus offering a unique platform to investigate in vitro tissue-cell responses to different exogenous insults. 

We hence performed ex vivo experiments on non-invasively collected biological materials, i.e. whole blood and skin biopsy slices. Samples were treated with three different contaminants, namely polybrominated diphenyl ether-47 (PBDE-47), mono-2-ethylhexyl phthalate (MEHP), and chrysene, and then examined by testing a set of biomarkers (in blood samples) or by applying a 2D-PAGE/MS-based proteomic approach (in skin slices).

While concerning blood samples we focused on the effects of treatments testing biomarkers of oxidative stress (total antioxidant status, “TAS”), immune system (respiratory burst, lysozyme, and complement system), and genotoxicity (erythrocytic nuclear abnormalities, “ENA”, assay), skin-biopsy slices were investigated by applying a holistic proteomic approach.

Blood is a fluid tissue that bathes almost all other tissues and organs. It is crucial in the physiology of an organism, and its dysfunctions may have serious systemic effects. Testing specific biomarkers in blood samples directly exposed to contaminants may therefore provide relevant data to understand the systemic effect of the investigated toxicants.

On the other hand, the proteomic approach would lead to the identification of a protein-biomarker panel characterizing the sample response to pollutant exposure in *C. caretta*. As proteins are the molecular effectors of biological systems, and their dynamics define physiological as well as pathological states, the effects of contaminants on the skin proteomic profiles may have, in reason of direct and prolonged contact of this tissue with toxicants present in sea water, a consistent relevance from an ecotoxicology perspective.

The ex vivo approach we proposed, combined with biomarker assay and proteomic investigation, may therefore represent a precious step forward in understanding the impact the tested contaminants may have on *C. caretta*.

## 2. Materials and Methods

### 2.1. Sample Collection

The tissues-collection procedure was carried out in strict accordance with the relevant national and international guidelines under CITES permits (CITES Nat. IT025IS, Int. CITES IT 007). Approval of the research study was obtained from the University of Siena and the museum of “Accademia dei Fisiocritici” (which is an institution in Siena devoted to releasing the permits).

In 2018, eleven specimens of loggerhead turtle adults, far from the reproductive period, were sampled in the Centro Recupero Tartarughe Marine of Manfredonia (Puglia, Italy). Once arrived at the rescue center, each loggerhead sea turtle was examined for the presence or absence of injuries (fishing nets, malnutrition, boat strikes, skin diseases) and, when stabilized, the sampling collection started. Morphometric parameters were registered. The blood samples (2–6 mL) were obtained from the dorsal cervical sinus of 7 specimens using a disposable syringe and transferred to solvent-rinsed glass vials with Teflon caps containing heparinized saline (heparin sodium). From another 4 specimens, a skin biopsy (20 mg each) was collected, using a biopsy punch with a 0.8 mm diameter, and divided in homogeneous sections (slices). Blood and skin biopsies were not taken from the same specimens by decision of the veterinarian.

### 2.2. Treatment Reagents

The compounds used for the experiment, 2,2′,4,4′-Tetrabromodiphenyl ether (BDE-47, 100% purity, CAS: 5436-43-1), phthalic acid mono-2-ethylhexyl ester (MEHP, 97% purity, CAS: 4376-20-9), and chrysene (98.4% purity, CAS: 218-01-9), were purchased from Sigma Inc. (St. Louis, MO, USA). All the treatments were diluted in dimethyl sulfoxide (DMSO, >99.5% purity, CAS:67-68-5) that was purchased from Sigma Inc. The culture medium consisted of Roswell Park Memorial Institute (RPMI) 1640 Medium and fetal bovine serum (FBS) 10% from Sigma lnc. (St. Louis, MO, USA). 

### 2.3. Tissue Exposure

For each animal, two whole-blood aliquots or two biopsy sections were exposed to three different contaminants: (*i*) chrysene (44 μM), a PAH consisting of four fused benzene rings; (*ii*) MEHP (100 μM), the ester of phthalic acid and 2-ethylhexanol; and (*iii*) PBDE-47 (10 μM), one of the 209 PBDE congeners characterized by two diphenyl rings, using DMSO 0.5% (*v/v*) as vehicle. Blood or skin aliquots were exposed also to DMSO 0.5% (*v/v*) only and considered as controls. We selected compounds belonging to classes of contaminants of particular concern for *C. caretta* in the natural environment and at the same time compounds that do not require to be bio-transformed to exert their toxic potential. Chrysene was demonstrated to induce dioxin-like [41] and estrogenic responses [42] in in vitro experiments. PBDE-47 is able to show immunomodulatory activity in fibroblastic cell lines from fish by increasing intracellular reactive oxygen species formation [43], activating apoptotic pathways in in vitro macrophages from mice [17], and releasing pro-inflammatory cytokines in porcine alveolar macrophage culture [44]. MEHP is a primary metabolite of the di-2-ethylhexyl phthalate (DEHP), and several studies used MEHP as a model compound to investigate the toxicity of phthalates [45]. The above-mentioned doses of the applied compounds were selected according to previous studies, where they were demonstrated causing sub-lethal effects without damaging tissue cells [16,46].

Each blood aliquot was diluted 1/5 in the culture medium containing contaminants and/or DMSO (final total volume 1 mL). The biopsies (8 ex vivo slice cultures from each enrolled animal were obtained and tested two by two) were maintained in 1 mL of the same mixture used for the blood. Samples were incubated at 25 °C for 24 h on a tilting platform. After the exposure time, whole blood was used for blood smears, an aliquot was frozen, and the remaining was transferred in plastic tubes and centrifuged for 5 min at 5000× *g* to obtain plasma. After 24 h, the skin biopsies were removed from the mixture, and for each animal, the slices pairs treated with the same contaminant were then prepared, keeping them together for the proteomic analysis. The same procedure was performed for the two control DMSO slices. All the biological materials were stored at −80 °C until analysis and the smears dried to air.

### 2.4. Sample Preparation for 2D-PAGE

Frozen skin slices from ex vivo cultures exposed to the tested contaminants were reduced into smaller pieces by using a scalpel to facilitate their homogenization. Pieces from the same treated sample were then homogenize in 400 µL of a conventional extraction buffer for 2D-PAGE analysis (8 M urea, 1% (*w/v*) DTE, 0.4% (*w/v*) CHAPS) and by using a TissueLyser II (TL) (Qiagen). An iron bead, 5 mm in diameter, was added in each TL tube for improving mechanical disruption of the sample. Teflon tubes and iron beads were frozen before their use at −80 °C to reduce sample heating. Due to the very leathery nature of the *C. caretta* skin, several steps of TL agitation were needed for a proper protein extraction. In brief, samples were agitated 5 min at 30 F for 6 times, with 10 min interval between each agitation step. During intervals, samples were cooled down by placing them at –80 °C. In addition, after 3 cycles of shaking and pause, samples were centrifuged at 3× *g* for 30 min at 4 °C to reduce sample–solution foaming. Then, after removing beads, samples were centrifuged for 15 min at 20.817× *g* at 4 °C. Resulting pellets were thoroughly pounded with a tube dounce, in ice, to increase sample solubilization. After a further centrifugation step of 20 min at 20.817× *g* at 4 °C, supernatants were recovered, and protein estimation was performed according to the Bradford method [47]. 

### 2.5. 2-DE and Image Analysis

Two-dimensional electrophoresis was performed using the Immobiline-polyacrylamide system. Isoelectric focusing (IEF) was carried out on a non-linear precast pH gradient from pH 3 to 11, 18 cm in length (Cytiva, formerly GE Healthcare, Uppsala, Sweden) and performed using the Ettan™ IPGphor™ system (Cytiva). Strips for analytical and mass spectrometry (MS) preparative runs were rehydrated overnight in 350 µL of the 2D-PAGE lysis buffer described above, and sample cup-loading was performed. Then, 60 and 600 µg of total protein were loaded for analytical and preparative gels, respectively. Bromophenol blue in trace was added to the lysis buffer and used for strip rehydration and to load sample solutions. In these latter, carrier ampholytes (pH 3–11) were present at 0.2% (*v/v*) for analytical runs and at 2% (*v/v*) for MS-preparative runs. IEF was performed on an Ettan IPGphor Manifold (Cytiva) at 16 °C and according to the following voltage steps: 200 V for 8 h, from 200 V to 3500 V in 2 h, 3500 V for 2 h, from 3500 V to 5000 V in 2 h, 5000 V for 3 h, from 5000 V to 8000 V in 1 h, 8000 V for 3 h, from 8000 V to 10,000 V in 1 h, and 10,000 V until a total of 93,000 Vh was reached. 

After IEF, the immobilized pH gradient (IPG) gels were equilibrated in 6 M urea, 30% (*v/v*) glycerol, 2% (*w/v*) SDS, 0.05 M Tris–HCl pH 6.8, 2% (*w/v*) DTE for 12 min, and for further 5 min in 6 M urea, 30% (*v/v*) glycerol, 2% (*w/v*) SDS, 0.05 M Tris–HCl pH 6.8, 2.5% (*w/v*) iodoacetamide, and trace of Bromophenol blue.

The second dimension was carried out at 9 °C on 9–16% (*w/v*) polyacrylamide linear gradient gels at 40 mA/gel constant current. Analytical gels were stained with ammoniacal silver nitrate according to Oakley et al. [48], while MS-preparative gels were stained according to the Sinha et al. [49] MS-compatible silver staining protocol.

Stained gels were digitized using the Image Scanner III coupled with the LabScan 6.0 software (Cytiva). Image analysis was carried out using ImageMaster 2-D Platinum 7.0 software (Cytiva).

The four gel classes, i.e. chrysene-class, MEHP-class, PBDE-47-class, and DMSO-class, were delineated by including in the same class all the gels from the same treatment. An intra-class analysis was then performed for each treatment class by matching all gels of the same class with their reference master gel. In order to detect quantitative and qualitative differences, a differential inter-class analysis was then done by comparing the four master gels. The relative volume (% Vol) values (integration of optical density of a single spot (volume) divided by the total volume of spots and expressed as a percentage) were assumed as the evaluation parameter.

### 2.6. Biomarker Analysis in Blood

Before starting the biomarker analysis, the cell viability test was performed on all blood samples using the trypan blue dye exclusion technique.

#### 2.6.1. TAS

A commercial kit (Antioxidant Assay Kit, Sigma, St. Louis, MO, USA) based on the method of Miller et al. [50], modified as follows, was used to evaluate the TAS. A stock solution of 1.5 mM of Trolox, a water-soluble analogue of vitamin E, was used for the standard curve. Trolox was diluted in assay buffer for the preparation of the different standard curve points (0, 0.015, 0.045, 0.105, 0.21, 0.42 mM). Aliquots of each concentration (10 μL/well) were added to a 96-well plate in duplicate. For each sample, 30 μL of plasma diluted 1:100 in assay buffer was added in duplicate to the plate. To each well, 20 μL of myoglobin as added followed by 150 μL of chromogen (ABTS (2,2-Azino-di [3-ethylbenzthiazoline])), and the plate was incubated at room temperature for 4.5 min. Absorbance at 405 nm was measured using a microplate ELISA reader (Microplate Reader Model 680XR, Bio-Rad). The TAS was expressed as mM of Trolox by linear regression of the standard curve.

#### 2.6.2. Respiratory Burst

The respiratory burst activity was evaluated as the presence of intracellular oxyradical produced by NADPH oxidase, and it was measured with the Nitroblue Tetrazolium (NBT) assay following the method of Secombes [51], modified as follows. For each sample, 100 μL of whole blood was added in triplicate to a 96-well plate and incubated at 30 °C for 2 h to allow cell adhesion. Unattached cells were then washed off 3 times with L-15 medium. To each well, 100 μL of L-15 medium supplemented with NBT (1 mg/mL) was then added, and the plate was incubated at room temperature for 1 h. After incubation, the plate was discarded and fixed with 100% methanol for 10 min. The plate was washed several times with 70% methanol and air-dried. To each well, 120 μL of KOH and then 140 μL of DMSO were added in order to destroy the cell wall and dissolve the crystals of formazan blue deriving from the reduction of NBT by the oxyradicals. Measurements were performed at 630 nm using an ELISA microplate reader (Microplate Reader Model 680XR, Bio-Rad) using KOH/DMSO as white. The respiratory burst activity was expressed as a reduction of NBT.

#### 2.6.3. Lysozyme Activity

The lysozyme activity was measured in plasma samples with a standard turbidity test described by Keller et al. [52], modified. Briefly, 1 mg/mL stock solution of hen egg white lysozyme (HEL, Sigma, St. Louis, MO, USA) was prepared in 0.1M phosphate buffer (pH 5.9) and serially diluted in phosphate buffer to produce the standard curve of 0, 0.3, 0.6, 1.25, 2.5, 5, 10, 20, and 25 μg/mL. To obtain a fresh solution of *Micrococcus lysodeikticus* (Sigma, St. Louis, MO, USA), 50 mg of the lyophilized cells were dissolved in 0.1 M phosphate buffer. Each concentration of the standard curve (25 μL/well) was added to a 96-well plate in triplicate, and 25 μL of each sample was added in quadruplicate to the same plate. The *M. lysodeikticus* solution (175 μL/well) was quickly added to three sample wells and to each of the standard wells. The blank was the fourth well containing plasma with 175 μL of phosphate buffer without *M. lysodeikticus*. Absorbance at 450 nm was measured using a microplate ELISA reader (Microplate Reader Model 550, Bio-Rad). The optical density (O.D.) was measured immediately (T0) and after 5 min (T5) and the activity expressed as HEL concentration (μg/mL) by linear regression of the standard curve.

#### 2.6.4. Serum Hemolytic Complement Assay

Serum complement activity was determined by the method of sheep red blood cells (SRBCs) hemolysis following the protocol of Mayer (1961) [53] and modified by Merchant and Britton [54]. In brief, heparinized whole blood collected from a healthy Merino sheep (*Ovis aries*) at a private sheep farm was centrifuged at 3000× *g* to obtain fresh SRBCs. Then, the SRBCs were washed with phosphate-buffered saline (PBS, pH 7.4) several times until supernatant was clear and diluted to 2% (***v/v***) in PBS. Turtle plasma was then incubated with an equal volume of 2% SRBC (***v/v***) for 30 min at 25 °C for 30 min. Thereafter, the sample was centrifuged at 2500× *g* for 5 min. To measure the optical density (O.D.), 300 µL of supernatant were used in microplate reader at 540 nm (Perkin Elmer Victor Nivo Microplate Readers). As a positive control, 2 µL Triton X-100 was added to 700 µL of a 1% SRBC suspension and homogenized until complete hemolysis; then, it was centrifuged and the optic density (O.D.) measured (considered 100% hemolysis). The results were expressed as mean % hemolysis = (O.D. sample/O.D. positive control) × 100. 

#### 2.6.5. ENA Assay

Erythrocytic nuclear abnormalities (ENA) assay was evaluated on blood smears according to the method of Casini et al. [12], modified. The smears were stained with Diff-Quick, and 1000 mature erythrocytes per sample were scored into one of the following categories: micronuclei, lobed nuclei, segmented nuclei, and kidney-shaped nuclei. The results were expressed as the ENA frequency, the mean value (‰) of each abnormality, and the sum of all the lesions observed.

### 2.7. Statistical Analysis

Unsupervised analysis was carried out using all the (2141) matched 2-DE spots. In particular, Principal Component Analysis was used as overall quality control of individual and spot variabilities, whereas the Spearman correlation heatmap highlighted potential co-expression protein patterns among all matched spots. Similarly, the heatmap of % Vol values from all matched spots showed the protein overall trend across the treatment groups. The hierarchical tree clustering of the maps was built by Ward’s minimum variance method and Euclidean distance.

The 2-DE data from three treatment groups (MEHP, chrysene, and PBDE) were compared against the control (DMSO) cohort using non-parametric paired statistics. Spots presenting significance (*p*-value and Benjamini–Hochberg FDR < 0.05) at Kruskal–Wallis, significant *p*-value at Nemenyi multiple comparison post-hoc test, and a fold change of ±2 in at least one comparison were considered as differentially abundant.

Three dimension PCA analysis was assessed onto the 26 significant differentially abundant spots. 

Blood biomarker statistical analyses were performed on the seven animals. Data from the different treatment classes were compared by non-parametric paired statistics for multiple groups and the Friedman test combined with the Nemenyi one as well. FC was computed as for the 2-DE analysis. For visualization, blood biomarker values from each animal were then normalized on the corresponding control values (DMSO).

All the above-reported statistical analyses and corresponding figures were obtained by applying the R software (R Core Team, Vienna, Austria. https://www.R-project.org; accessed on 9 December 2021) [55].

### 2.8. Protein Identification by MALDI TOF/TOF 

Statistically significant differential spots were cut out manually from MS-preparative 2-DE gels. Spots were destained in a solution of 30 mM potassium ferricyanate and 100 mM sodium sulphate anhydrous until complete destaining. Then, spots were conditioned in a 200 mM ammonium bicarbonate solution for 20 min and dehydrated in 100% acetonitrile (ACN). Protein spots rehydration and digestion occurred overnight at 37 °C in a trypsin solution. Digested proteins were then spotted on a MALDI target and allowed to dry. A matrix solution of 5 mg/mL α-cyano-4-hydroxycinnamic acid (CHCA) in 50% (***w/v***) ACN and 0.5% (***v/v***) trifluoroacetic acid (TFA) was applied on each dried sample–peptide mixture and dried again. Mass spectra were acquired using an UltrafleXtreme ™ MALDI-TOF/TOF instrument equipped with a 200 Hz smartbeam™ I laser in the positive reflector mode (Bruker, Billerica, MA, USA). The following parameters were applied: 80 ns of delay; ion source 1: 20 kV; ion source 2: 18.85 kV; lens voltage: 9.50 kV; reflector voltage: 26.30 kV; and reflector 2 voltage: 14.00 kV. The applied laser wavelength and frequency were 353 nm and 100 Hz, respectively, and the percentage was set to 50%. Final mass spectra were produced by averaging 1500 laser shots targeting three different positions within individual sample–peptide mixtures spotted on the target. FleXanalysis software version 3.4 (Bruker) was used to analyze mass spectra and for assigning peaks. Acquired spectra were calibrated using peptides arising from trypsin autoproteolysis as an internal standard. The resulting mass lists were then cleaned from contaminant peaks as matrix-related ions and trypsin autolysis. Peptide Mass Fingerprinting (PMF) search was performed using MASCOT (Matrix Science Ltd., London, UK; http://www.matrixscience.com; accessed on 15 December 2021) setting up the following parameters: Chordata as taxonomy, UniProtKB/SwissProt and NCBIprot as databases, 50 ppm as mass tolerance, one admissible missed cleavage site, carbamidomethylation (iodoacetamide alkylation) of cysteine as fixed modification, and oxidation of methionine as a variable modification. Tandem mass spectra were acquired using the LIFT mode, and the same previous search parameters were applied to MS/MS ions search in MASCOT plus 0.1% peptide tolerance, 0.5 Da MS/MS tolerance, and positively monocharged ions. MS analyses were performed at the Molsys Technology Platform (http://molsys.dbcf.unisi.it; accessed on 12 December 2021).

## 3. Results and Discussion

The several ethical and physical limits in exposing large, long-lived, and threatened species such as sea turtles to pollutants, which could compromise their wellness and survival, have significantly reduced our capability in understanding and assessing the effect that contaminants may have on these animals. Previous works have tried to bypass such issues by taking advantage of cell lines or primary cultures obtained from different specimens [27,28,56]. Cell cultures represent a valid approach for toxicity bioassay; on the other hand, they do not entirely reproduce cell heterogeneity and acellular structures that define functional properties and characteristics of a tissue or an organ.

In this study, we sought to improve the similarity of the in vitro to the in vivo system by using an ex vivo approach to obtain an accurate evaluation and prediction of the skin tissue and whole-blood biochemical and cellular responses to direct contaminant exposure.

### 3.1. Proteomic Characterization of Chrysene, MEHP, and PBDE-47 Effects on Ex Vivo Skin-Samples

**Figure 1:**
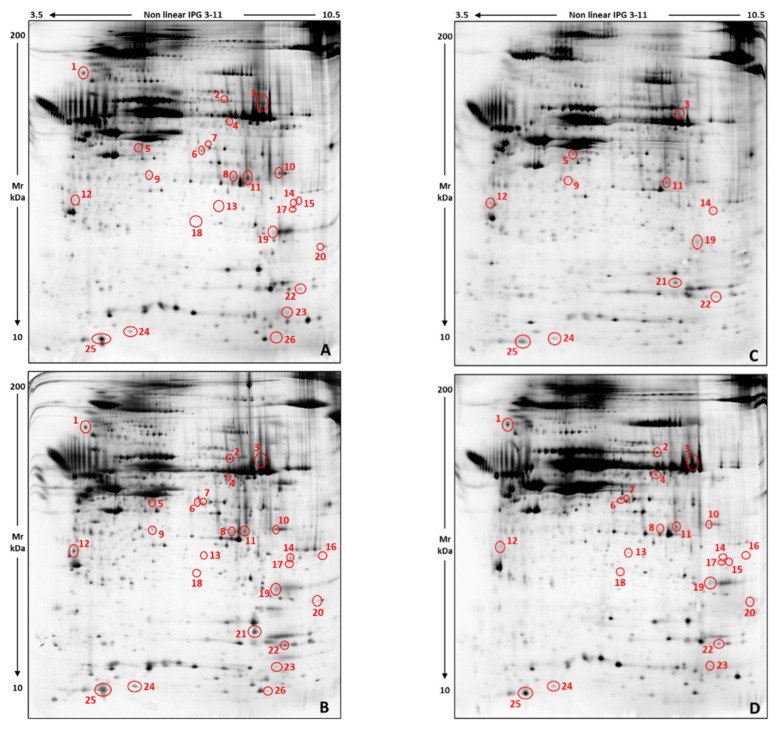
Representative 2-DE protein patterns of ex vivo skin-slice cultures from adult *C. caretta* turtles, treated with the DMSO vector (i.e. Ctrl) (**A**), MEHP + vector (**B**), chrysene + vector (**C**), and PBDE-47 + vector (**D**). Red circles and numbers evidence differentially abundant protein spots detected by comparing Ctrl vs. chrysene, MEHP, and PBDE-47; chrysene vs. MEHP and PBDE-47; and MEHP vs. PBDE-47. Numbers correspond to those listed in Table 1 and in Supplementary Table S1.

Slice cultures of skin biopsies obtained from four different specimens of loggerhead sea turtle were individually exposed to chrysene, MEHP, and PBDE-47 by using DMSO as the contaminant vehicle. Skin-slice cultures maintained in DMSO alone were used as controls. From each enrolled animal we obtained 4 slice-culture samples and from each of them a 2-DE gel. The resulting 16 gels, 4 per each treatment, were digitized and the resulting images subjected to qualitative and quantitative image analysis by applying the ImageMaster software. Representative 2D gel images, one for each condition, are shown in Figure 1.

The four treatment classes were hence compared to each other and, by applying rigorous statistics, we detected 26 significant differences showing a minimum of two-fold abundance change between at least two of the experimental classes (Supplementary Table S1). The majority of significant differences occurred among chrysene and the other three treatments: chrysene vs. DMSO = 16 differences, chrysene vs. MEHP = 17 differences, and chrysene vs. PBDE-47 = 20 differences. They principally corresponded to protein spots detected in DMSO, MEHP, and PBDE-47 but not in 2D gels of skin slices exposed to chrysene. We also detected three protein abundance differences between MEPH and DMSO, four between PBDE-47 and DMSO, and seven between MEHP and PBDE-47. In Figure 1, significant differences are evidenced by red circles and numbers (Table 1 and Table S1). 

**Figure 2:**
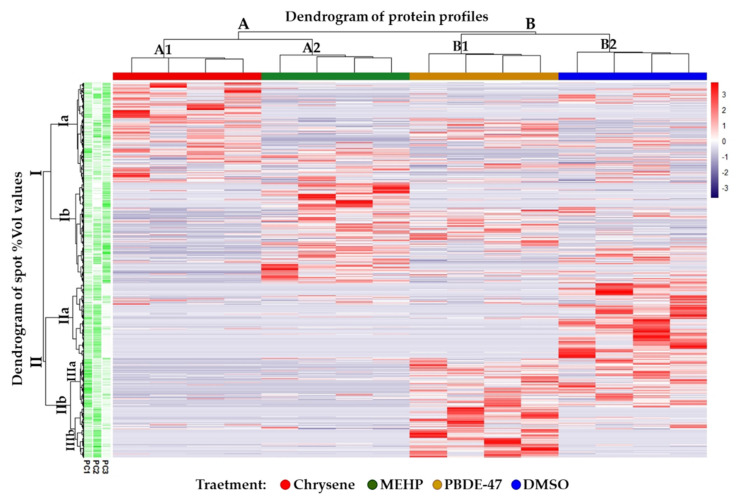
Spot intensity Heatmap. Tree clustered spot-intensity heatmap performed on all the spots. In column annotations, chrysene, MEHP, PBDE-47, and DMSO groups are represented in red, green, yellow, and blue, respectively. The row annotation gradients reported the absolute contributions of each spot in the first three Principal Components (PCs).

Our preliminary ex vivo study highlighted specific changes of the skin-slice protein profiles caused by individually tested pollutants, which were used at non-cytotoxic concentrations and for a short-term exposure. As highlighted by the spot-intensity heatmap in Figure 2, specific protein profiles were clearly delineated for each toxicant class despite protein-abundance fluctuations in individual animals. The dendrogram on the left of the heatmap, i.e. “dendrogram of spot % Vol values”, allows an immediate evaluation of four main spot clusters, which roughly corresponds to proteins defining the individual contaminant signature on the skin-slice proteome of *C. caretta*. The spot clusters Ia and Ib, except for some overlapping % Vol values in a sub-cluster from Ia, distinguish chrysene from MEHP. On the other hand, the spot cluster IIa differentiates DMSO from the other three treatments, while the sub-cluster IIIb from the cluster IIb characterizes the PBDE-47 class. The general spot-abundance trends from individual ex vivo skin cultures, differentially treated, clustered by similarity into four distinct groups (i.e. A1, A2, B1, and B2) that exactly correspond to the four treatment classes, as stressed by the “dendrogram of protein profiles” on the top of the matrix. In general, chrysene and MEHP clusters showed a protein pattern more similar to each other and quite different from that of the PBDE-47 and the control. In fact, they diverge from cluster A of the protein profile dendrogram, while PBDE-47 and DMSO do from cluster B. In order to evaluate how delineated spot clusters correlate to the variance-covariance among protein patterns, we also report in Figure 2 the absolute contributions of each spot in the first three Principal Components (PCs) of the Principal Component Analysis (PCA) that we performed as follows.

**Figure 3:**
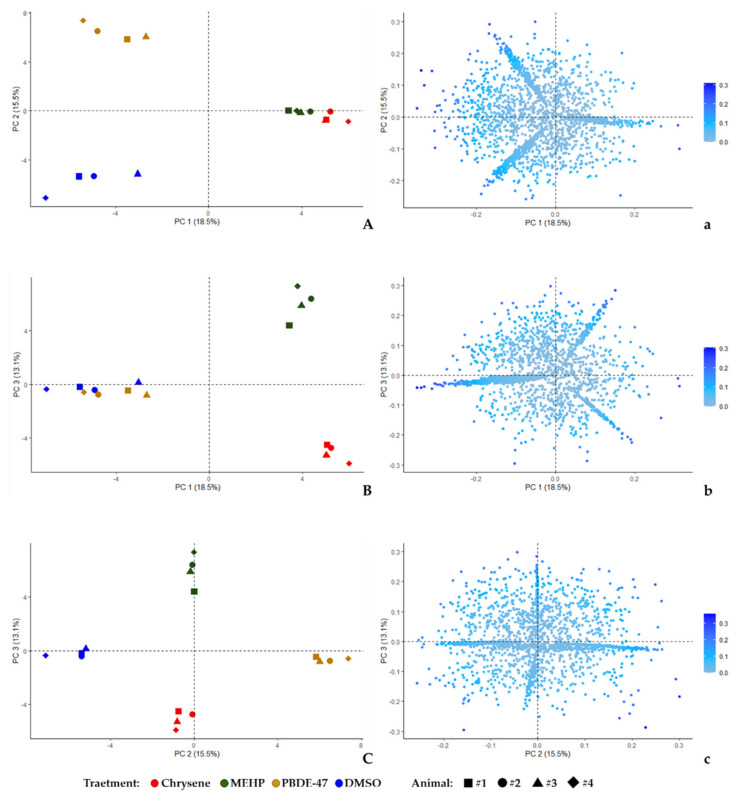
Unsupervised PCA. Principal Component analysis (PCA) plots performed on all the spots detected throughout the analyzed 16 gel images, 4 per tested condition. Three axonometries are shown combining the first three PCs: PC1 vs. PC2 (**A**), PC1 vs. PC3 (**B**), and PC2 vs. PC3 (**C**). Individual % values of explained variations are reported in axis for each PC, i.e. PC1 = 18.5%, PC2 = 15.5%, and PC3 = 13.1%. Within the same plot as well as in different plots, identical geometrical shapes indicate the same animal (square = animal #1; circle = animal #2; triangle = animal #3; rhombus = animal #4). The vector DMSO and the different contaminants, to which biopsies were exposed in combination with the vector, were instead represented by the color of the geometric shapes: chrysene = red; MEHP = green; PBDE-47 = yellow; and DMSO = blue. Variable plots (**a**–**c**) are reported in the same axonometries in which the % contribution of each spot to the given PCs is referred to as the blue gradient color scale.

**Figure 4:**
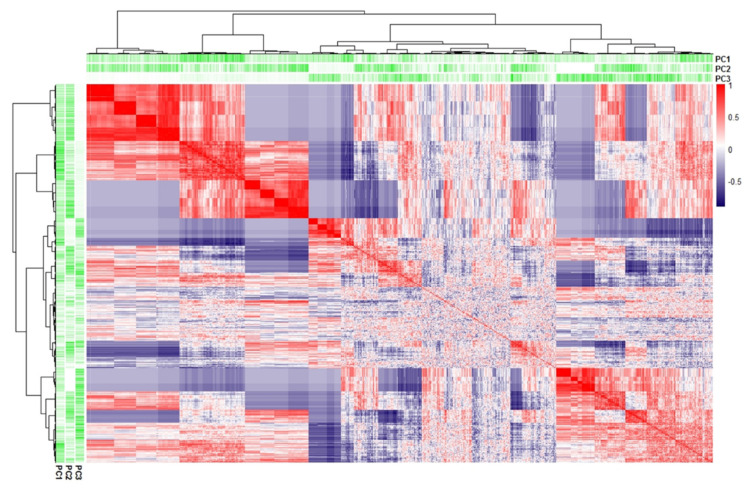
Correlation matrix of spot intensities. Tree clustered Spearman correlation matrix performed on all the spots. The gradient scale reported the Spearman coefficient (red as directly correlated, blue as inversely correlated, and white as not correlated). The column and row annotation gradients reported the absolute contributions of each spot in the first three Principal Components (PCs).

The unsupervised PCA on the spot % Vol values of all the matched 2-DE spots was performed to evaluate the variance-covariance occurring among protein patterns from the four tested conditions. The first three Principal Components (PC) summarized the 47.1% (PC1 = 18.5%, PC2 = 15.5%, and PC3 = 13.1%, respectively) of variance of the whole 2-DE dataset. The individual PCA plots show the segregation of chrysene and MEHP from DMSO and PBDE-47 clouds mainly occurring alongside the PC1, while PBDE-47 and DMSO separate throughout the PC2 and MEHP from chrysene by PC3 (Figure 3). The combination of PC2 and PC3 clearly divides the four groups and, at the same time, sharply clusters the four animals in relation to the treatment class. Despite the presence of partial overlapping features between DMSO and PBDE-47 (PC1) or between MEHP and chrysene (PC2), the correlation matrix (Figure 4) supports the spot-intensity heatmap by highlighting that each treatment stands out from the others according to individual protein-expression modules, which present specific protein abundances and characteristic protein correlations. In particular, the distinguishing protein patterns occurred on PC1 and PC2 for PBDE-47 alongside PC3 for MEHP, mainly on PC3 and PC1 for chrysene and on PC1 and PC2 for DMSO groups.

Furthermore, the spot-intensity correlation matrix combined with the absolute variance contributions of each spot in the first three PCs highlights an evident spot clustering based on direct or indirect correlation of spot % Vol in the different tested conditions (Figure 4). This further clarifies that different treatments have specific effects on the skin-slice protein profile and that such effects are detectable by our ex vivo approach, which indeed results as promising for ecotoxicological assessments in *C. caretta*.

In order to evaluate the biological relevance they may have in the exposed samples, we then attempted the identification of protein differences by using MS. *C. caretta* does not have a completed genome project. As expected, almost all the identifications were obtained in phylogenetically correlated species, in particular in *Chelonia mydas*, whose genome draft was released in 2013 [24]. We also obtained an inter-species identification in *Cariama cristata* (spot n. 5), a large South American bird also known as Red--legged Seriema. This is in line with previous genome drafts of *Pelodiscus sinensis* and *Chelonia mydas* indicating the close relationship of the turtles to the bird–crocodilian lineage [24]. In total, we identified 10 protein spots corresponding to 8 unique proteins (Table 1).

**Figure 5:**
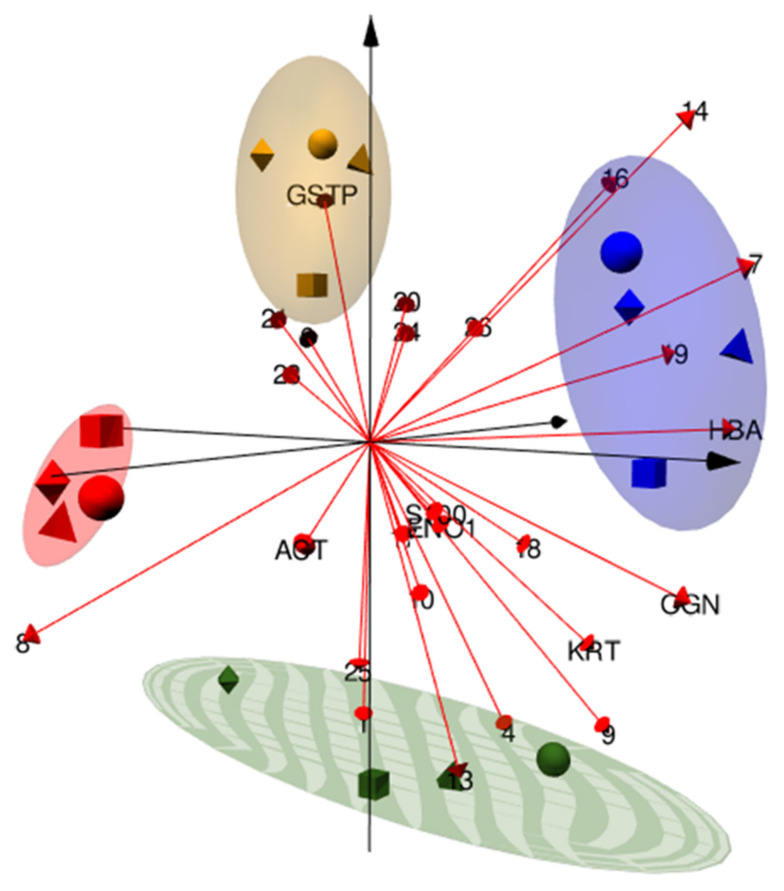
Differentially abundant spot PCA. 3D Principal Component analysis (PCA) bi-plots performed on the significant differentially abundant spots; 66.2% of variation is explained by the first three PCs (35.8%, 19.9%, and 10.5%, respectively). While the same geometrical form always indicates the same animal (square = animal #1; circle = animal #2; triangle = animal #3; rhombus = animal #4), different colors of the geometrical forms specify the different treatments: chrysene = red; MEHP = green; PBDE-47 = yellow; and DMSO = blue. Variables are illustrated as red arrows, and corresponding protein abbreviations (i.e. protein spots identified by MS) or spot numbers (i.e. not identified protein spots) are reported on their top. The 0.75 of confidence intervals per each cohort is shown by ellipses.

In Figure 5, the differentially abundant spot 3D PCA were reported showing the individual variance-covariance contribution throughout the first three PCs of detected protein differences to the different treatment classes delineation and reciprocal divergence. In the plot, identified differences are indicated by the corresponding gene name.

Among the obtained identifications, glutathione S transferase P (GST-P) is known in ecotoxicology and is used as a biomarker of contaminant exposure. Conversely, the other proteins we identified have never been described as participating in *C. caretta* tissue response to the contaminants we tested. However, they represent a starting point to clarify the biochemical and molecular mechanisms underlying the toxicity of the analyzed pollutants. In particular, the proteins mimecan, peptidyl-prolyl cis-trans isomerase A (PPIA), and protein S100A6 (S100A6) have numerous cellular functions whose alteration could compromise the health of the sea turtle. They should therefore be further investigated as new potential biomarkers in *C. caretta* ecotoxicology. 

#### 3.1.1. Glutathione S Transferase P

Glutathione S-transferase is a phase II enzyme that catalyzes reactions between glutathione and a variety of electrophilic compounds, including some environmental pollutants, thus protecting cells from oxidative stress. Glutathione conjugation to xenobiotic compounds also increases their water solubility and excretion or their further metabolization. Sinaei and Zare [36] described a significant correlation between total PAHs and GST activity in blood samples of *Chelonia mydas* from the Iranian coastline. However, no studies report on the direct effects of chrysene on sea turtle GST-P.

In the chrysene gel class of our proteomic study, we observed a consistent reduction of the GST-P proteoform from the spot 19 when compared to the other analyzed classes although its down regulation was not statistically significant in the chrysene vs. MEHP comparison (Figure 1 and Table 1). At present, we cannot exclude that chrysene or the other tested pollutants may induce changes in post-translational GST processing that may cause a pI and/or MW shift of the corresponding proteoform(s), which may also represent a tissue specific response to a short-term exposure to chrysene.

Conversely to the chrysene effect, the slight and not statistically significant downregulation of the spot 19 GST-P proteoform in the MEHP gel class suggests a MEHP toxic effect, compared to the control class, in reducing GST abundance. The DEHP bioactive metabolite MEHP is a potent endocrine-disrupting chemical (EDC) whose several health-threatening effects have been associated with its detrimental activity on the cell redox homeostasis [57]. Namely, it reduces glutathione (GSH) availability and interferes with the expression of GSH-related genes, such as the mammalian GST-Pi [58].

Among the wide range of toxic effects that it has on humans and wildlife, PBDE-47 causes oxidative stress by inducing an overproduction of reactive oxygen species (ROS) and mitochondrial dysfunctions [59,60]. In general, PBDE-47 induces the activation of xenobiotic detoxification systems although its lengthened exposure was described as inhibiting metabolic detoxification [61]. As a matter of fact, GST activity was found significantly related to PBDE-47 bioaccumulation in different aquatic species [61]. The % Vol increase of the spot 19 from the PBDE-47 gel class confirms the impact that this contaminant has on biological systems and how these attempt to counterbalance its injuries. In particular, the GST-P modulation we observed proves the reliability and the relevance of the skin ex vivo model, combined with the proteomics approach, for chemical risk assessment in ecotoxicology.

#### 3.1.2. Mimecan

Originally isolated from bovine bone [62], mimecan/osteoglycin (OGN) is a small leucine-rich proteoglycan, fundamental in collagen fibrillogenesis and in cellular growth, differentiation, and migration control [63]. Its aberrant expression was described as occurring in several human and other mammal disorders, from cardiovascular risk [64] to hypothalamic–pituitary–adrenal (HPA) axis dysfunctions [65], from obesity and related co-morbidities [66,67] to tumorigenesis [68], cancer [69,70,71], and bone homeostasis defects [72].

Different mimecan proteoforms exist. They result from alternative splicing and differential maturation of the protein precursors, often related to tissue specificity [63]. Interestingly, the mimecan proteoform profile in *C. caretta* ex vivo skin cultures were evidently affected by the three contaminants we tested. Three protein differences were identified as mimecan, and an overall decreasing trend, compared to the control, was observed in all of them, with chrysene exposure resulting as the most deleterious (Table 1: spots 8, 10, and 11). The only exception to this decreasing trend was represented by the MEHP induced upregulation of the spot 10, which in any case demonstrates a dependent contaminant modulation of the mimecan proteomic pattern. Different proteoforms have different physico-chemical properties, and affecting their reciprocal occurrence in a tissue may in fact impact its physiology. In the literature, there is no article reporting the mimecan presence and function in sea turtles, and corresponding coding sequences are available (or annotated as mimecan/osteoglycin) only for *C. mydas* and *Dermochelys coriacea*, as proven by the NCBI gene tool search for the existing seven sea turtle species (https://www.ncbi.nlm.nih.gov/gene; accessed on 20 December 2021). However, mimecan genomic structure as well as its protein sequence is highly conserved among species, thus suggesting it acts in relevant and similar roles throughout species [73]. Although we can only speculate on its physiological functions as well as on its involvement in pathogenesis of disorders affecting these marine reptiles, mimecan modulation may reasonably concur to the previously described detrimental effects that our tested toxicants have on different tissues and related systems also in sea turtles. In fact, we may suppose that these contaminants change the proteoform profile of mimecan even in other tissues although in a tissue-dependent manner, where specific mimecan functions have been described in other species and whose dysregulation may concur to the toxicant exposure effects.

Noteworthy, in mimecan-deficient mice, the skin mechanical strength is consistently reduced [73], and in humans, its decreased presence was suggested as a marker of epithelial to mesenchymal transition [70,74]. In particular, mimecan was reported as downregulating HIF-1α activity and inhibiting VEGF synthesis, recruiting CD8+ T cells, and controlling autophagy in human colon cancer [69]. Fibropapillomatosis (FP) is a debilitating complex disease affecting all the sea turtle species worldwide. It is characterized by the development of single or multiple cutaneous and internal tumors that debilitate and have the potential to kill affected animals. Although herpesviral etiology has been principally recognized for it [75], multiple factors seem to participate in FP development [76] and progression, and a defective mimecan profile may indeed represent a risk factor in its pathogenesis, at least in skin.

Chrysene is known for causing chromosome aberration in rodents [77] and was classified as a possible human carcinogen (group 2B) by the International Agency for Research on Cancer [78]. As observed in in vitro analysis using pig skin and the HaCaT keratinocyte cell line, its penetration and permanent retention in skin may cause cutaneous toxicity resulting in keratinocyte activation, COX-2 and PGE2 upregulation, and inflammation [79]. Among the contaminants we tested, chrysene causes the most evident effects on the mimecan profile in skin by downregulating all the mimecan isoforms we identified. Chrysene exposure could hence have a negative role in *C. caretta* skin defects and maybe also in fibropapillomatosis.

In the human ovary, the highest relative expression of mimecan was observed during a critical ovarian developmental stage, i.e. when oogonia differentiate in oocytes [80]. In addition, it was reported upregulated in ovine granulosa cells from large follicles [81] and increased during geese follicular development [82]. An eventual change in mimecan expression and proteoform pattern may hence have a negative effect during oocyte formation. Despite its hydrophobic nature, in humans chrysene can spread throughout the body, transported by the bloodstream, and reach the ovary and the follicles [83]. Sinaei and Zare [36] demonstrated the presence of chrysene in the blood of *C. mydas* from different areas, and its accumulation was described in *Malaclemys terrapin* eggs from Mangrove Lake, Trott’s Pond, and South Pond in Bermuda [84]. Reasonably, ovary/follicular chrysene may interfere with mimecan presence and proteoform occurrence, which, in their turn, may influence oocyte development and quality.

MEHP is known to cause metabolic disorders, endocrine disruption, and development and reproductive impairments in humans and other species [57,85,86,87,88,89,90]. DEHP is rapidly metabolized, both in invertebrates and vertebrates, in its primary metabolite, MEHP [91]. Consequently, in DEHP- and MEHP-exposed sea turtles, MEHP may reach the ovaries through the circulation and probably affect, among others, the mimecan profile. Interestingly, a recent work described the accumulation of DEHP in *C. caretta* unhatched eggs [40], but no studies reported MEHP presence in the ovary or eggs of this species.

#### 3.1.3. Peptidyl-Prolyl Cis-Trans Isomerase

Peptidyl-prolyl cis-trans isomerase A, also known as cyclophilin A or rotamase A, is a ubiquitous and multifunctional protein enzymatically active in protein folding by catalyzing the cis-trans isomerization of proline imidic peptide bonds. PPIA belongs to the highly conserved protein family of cyclophilins [92] and is active in diverse biological processes that differentially contribute to cellular functions and tissue physiology [93]. Namely, PPIA enhances peroxiredoxins antioxidant activity [94,95] and inhibits apoptosis [96], regulates RNA transcription and maturation as well as mitochondrion homeostasis [97,98]. It controls leukocyte chemotaxis, T cell activation, and inflammatory cytokine release [99,100,101], and is active in cellular division [102], proliferation, differentiation, and migration [103,104,105].

As expected from its numerous functions, PPIA anomalies have been suggested to be involved in key processes underlying several human pathologies, including viral infections and protozoan parasitosis [106,107,108]. In particular, PPIA differential localization and post-translational modifications have been related to different pathological roles in a number of diseases [109].

According to our ex vivo analysis, PPIA % Vol variations resulted not significant in all the treatments when compared with the control. Nonetheless, we observed an upregulation trend in skin slices cultured in MEHP-added medium, and this trend resulted significant in the MEHP vs. chrysene comparison (Table 1: spot 22). Although Lu et al. [110] described a PPIA increase as a DEHP dose-dependent effect in *Venerupis philippinarum*, no work reports, in the literature, an MEHP-dependent increase or proteoform modulation of PPIA. Nonetheless, according to the several rules acted by this rotamese in protein folding, trafficking, and assembly as well as in cell signaling and immune modulation, the PPIA increase or proteoform-pattern variation may be considered a tissue response to the MEHP induced stress.

Interestingly, changing PPIA abundance or its post-translational modifications may be an alternative, not yet investigated process through which MEHP may influence the inflammatory and immune response in exposed organisms. In fact, some of the phthalate-affected pathways intersect those modulated by PPIA [45]. As observed in several human cancers induced by viruses, defective immune response may allow the viral load to increase up to an oncogenic threshold, beyond which tumor lesions develop. PPIA is known to either promote or inhibit virus replication by controlling cell infection, virus genome replication, and capsid stabilization and by regulating inflammation and immune response. As PPIA also controls cell proliferation and apoptosis, MEHP effects on the immune system and PPIA proteomic profile may impact on the susceptibility of sea turtles to the chelonid herpesvirus 5 (ChHV5) and on the fibro-epithelial lesions formation and development. As discussed above for mimecan, aberrant PPIA functions may hence represent a risk factor for FP onset and expansion. 

#### 3.1.4. Protein S100-A6

Protein S100-A6, also known as calcyclin, is a Ca2+-binding protein from the S100 protein family [111]. In adult organisms, it is a cell-type-specific protein whose expression is increased in numerous tumors [112,113,114,115]. It is, in fact, active in the control and progression of the cell cycle, in cytoskeleton organization and dynamics, in apoptosis regulation, and in inflammation induction [116]. In the proteome profile of the ex vivo MEHP-treated skin we identified S100A6 as a differentially abundant protein significantly increased in the MEHP vs. chrysene comparison. A trend of MEHP-dependent increase of S100A6 was also observed, although not significant, in the MEHP vs. control comparison. As stated above, MEHP induces ROS production in several and different tissues among species, and S100A6 is widely described as upregulated in response to stress conditions such as oxidative stress [117]. As a rule, the upregulation trend we observed may be considered an ex vivo tissue response to redox homeostasis perturbation (Table 1: spot 25). On the other hand, chronic exposure to MEHP could sustain S100A6 upregulation over time, with a reasonable risk of developing skin disorders. There is a lack of literature describing a correlation between exposure to MEHP and modulation of S100A6. Nonetheless, S100A6 has been described as associated with epidermal carcinogenesis [118], and according to its roles in promoting proliferation and tumor phenotype, S100A6 deregulation may represent a risk factor for FP onset and development.

### 3.2. Biomarkers in Whole Blood Treated with Chrysene, MEHP, and PBDE-47 

The ex vivo system proposed in this study, which uses whole blood, has never been applied until now in *C. caretta* or in other reptiles; the few studies concern humans [119,120] and monkeys [121]. The study by Ivanov et al. [119] proved the ability of the ex vivo system to respond to ionizing radiations with formation of chromosome aberrations, a genotoxicity endpoint, while the studies by Fischer et al. [120] and by Krakauer et al. [121] demonstrated the modulation of cytokines secretion in whole blood after exposure to different compounds. Cocci et al. [20] used erythrocyte cultures from *C. caretta* exposed to a phthalate, the DiDP. In the present study the cell viability of all blood samples indicated a good condition for the feasibility of the biomarker analysis, showing values higher than 85%. Whole blood was treated with three compounds relevant for *C. caretta* in the natural environment: chrysene, MEHP, and PBDE-47. We focused our attention on endpoints of particular interest for the evaluation of the effects of different contaminants, such as oxidative stress, immune system, and genotoxicity, because they are poorly investigated in this species and because an alteration of these biological responses can lead to a strong impairment of the health of the loggerhead sea turtles. The responses of the biomarkers, following exposure of whole blood to the different compounds, showed a certain variability also in the control values, and for this reason, the values of the treated samples were normalized as indicated in the Materials and Methods section.

#### 3.2.1. Total Antioxidant Status

The antioxidant system, enzymatic and non-enzymatic, represents the first line of defense against the excessive production of ROS, allowing to obtain more information on the antioxidant defenses. A high concentration of antioxidants indicates that the examined organism was probably exposed to contaminants that are able to cause oxidative stress. A low TAS level might indicate either absence of oxidative stress or a situation in which the organism has achieved a homeostatic level after overcoming an excessive ROS presence. In the present research, TAS showed average values of 8.59 mM, which proved to be in line with those observed in the paper of Caliani and collaborators (2019) [14], in which 61 blood samples from animals in rescue centers were analyzed. The results did not show a clear pattern in any of the treatment groups, with data varying almost uniformly around the control value, and no induction of oxidative stress was highlighted. In recent papers, Finlayson et al. [27,28] tested phenanthrene and other persistent organic contaminants in cell cultures of fibroblasts from and other tissues and did not find induction of oxidative stress. The results obtained together with the reported literature let us hypothesize that the absence of oxidative stress may indicate a highly effective defense mechanism.

#### 3.2.2. Respiratory Burst

The respiratory burst, or oxidative burst, is a defense mechanism of phagocytes, which produce large quantities of reactive oxygen species to eliminate pathogens. The increase of ROS can damage the body’s cells and tissues. Respiratory burst has been shown in different animal classes [122,123,124,125] including reptiles [126]. In the study of Rousselet et al. [127], the respiratory burst, the ability of phagocytosis, the activity of natural killer cells, and the proliferation of lymphocytes in the blood of 65 young specimens of loggerhead sea turtles were evaluated. The results showed a greater ability of monocytes to carry out respiratory burst and phagocytosis than other categories of leukocytes, and for the first time, the activity of natural killer cells was observed and measured, and lymphocyte proliferation was confirmed in response to presence of mitogens in the blood cells of *C. caretta*. Respiratory burst was also measured in whole blood in the same species [14], showing results in line, albeit slightly lower, than those found in this study. The respiratory burst, similar to the TAS, does not show clear response patterns in the different treatments although the treatment with MEHP shows a prevalence of samples with values higher than the controls.

#### 3.2.3. Lysozyme Activity

**Figure 6:**
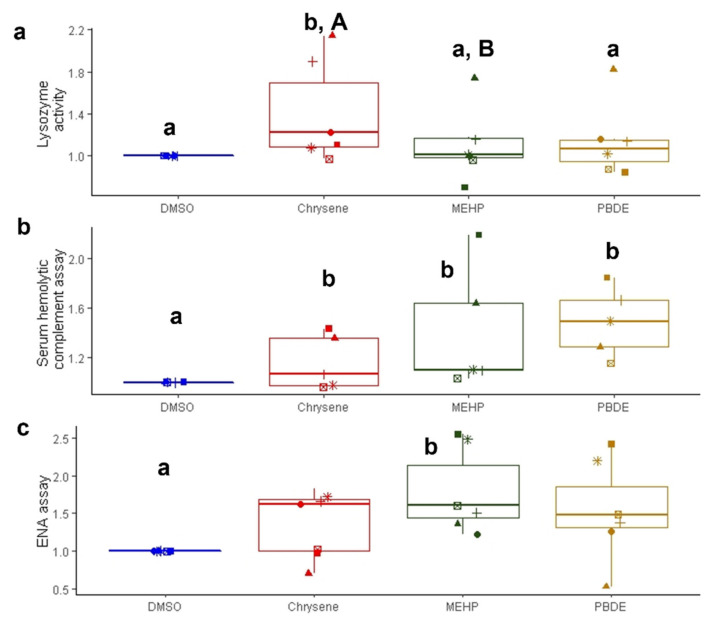
Lysozyme activity, serum hemolytic complement assay, and ENA assay: box plot of lysozyme activity (**a**), serum hemolytic complement assay (**b**), and ENA assay (**c**) detected in blood samples of seven *Caretta caretta* specimens. DMSO, chrysene, MEHP, and PBDE-47 groups are represented in blue, red, green, and yellow, respectively. The paired samples (same animal) are indicated with different symbols across the treatments. Individual values are normalized on the control (DMSO) value of each animal merely to improve the visualization of the results, as properly detailed in the Material and Methods section. Different normal lowercase (e.g. a and b) indicate significant differences with respect to control (*p* < 0.05); different uppercase letters (e.g. A and B) indicate differences among the treatments.

Lysozyme, an enzyme secreted by phagocytes, i.e. macrophages, monocytes, and heterophiles [128], is able to cause the lysis of bacteria through the hydrolysis of the cell wall. Since it is a marker of pro-inflammatory responses and has an antibacterial role in plasma, it can be considered a biomarker of innate immunity [129,130]. A statistically significant induction was found following the treatment with chrysene both compared to the control and compared to the group treated with MEHP (Figure 6).

Induction trends were found also with the two other compounds. Some authors have evaluated this biomarker in *C. caretta* plasma [14,52,131,132,133]; most of the reported values were similar to those found in the present work. No information is available regarding the effects of the three tested compounds on the immune system or specifically on lysozyme activity in *C. caretta*. The few existing studies on *C. caretta* lysozyme concern the effects of classes of contaminants different from the ones investigated in the present study. Keller et al. [52] observed a negative correlation between the activity of lysozyme in the plasma of the loggerhead sea turtle and the relative concentrations of 4,4’-DDE and chlordane; Day et al. [131] observed a positive correlation between lysozyme activity and Hg concentration in the blood of 70 loggerhead specimens sampled in 2001 and 2003 along the coasts of South Carolina, Georgia, and Florida. Considering other reptiles, Neuman-Lee et al. [134] exposed pregnant specimens of garter snakes (*Thamnophis elegans*) and their offspring to PBDE-47, finding effects on reproductive parameters and an increase in immunocompetence. Up to now, few authors have investigated the concentrations of chrysene in the blood of sea turtles [33,135], but no information is available on the possible effects of this compound on the immune system of this species.

#### 3.2.4. Serum Hemolytic Complement Assay

The complement system represents an important component of the innate immune response in vertebrates and invertebrates [136]. It consists of a set of proteins localized in the plasma, the activation of which initiates the inflammatory response, leads to the marking of the exogenous particulate material, and eliminates pathogens through the assembly of a multiprotein complex that destroys the membranes of microorganisms [54]. This biomarker was measured in other reptiles before [53,54] and slightly readapted in this study for application in *C. caretta*. An ability to significantly induce this response was found for all tested compounds with respect to controls (Figure 6); these data are extremely important also for the possibility that these compounds have to act in the natural environment in a synergistic way or at least as a summation, being with high probability present at the same time in the marine environment and in sea turtles. Some studies report an increase in the immune response following exposure to DEHP in accordance with the results obtained in this work. The study by Zhong et al. [137] reported the significant increase in the concentration of some proteins (C3, C5, and SC5b-9) that are part of the complement system in human blood that had been exposed to PVC materials containing DEHP. The study by Watanuki et al. [138], carried out on cell cultures of carp phagocytes (*Ciprinus carpio*), reported a significant increase in the phagocytosis capacity of cells exposed to a concentration of 100 nM of DEHP and a significant increase in the production of superoxide anion in cells exposed to a 1000 nM concentration of DEHP. Furthermore, the study by Wang et al. [139] reported a significant decrease in the phagocytosis capacity in cell cultures of carp neutrophils following exposure to DEHP.

Regarding the statistically significant increase in the activity of the complement system in the blood treated with PBDE-47, no studies have been found in the literature, either in turtles or in other reptile species, evaluating the effects of PBDE-47 on this biomarker although, as also reported above, effects of this compound on immune systems, manifested as altered splenic lymphocyte populations, increased susceptibility to infections, and the release of pro-inflammatory cytokines [140,141,142], were reported for other species [16,17,143].

Effects of PAHs on immune system in sea turtles are scarcely investigated; few papers showed that a chronic exposure to these compounds is not lethal, but it could compromise the general health status of the animals, making it more vulnerable to stressors, such as the occurrence of cutaneous fibropapillomatosis and ultimately cause cancer [33,144]. The biomarker results (lysozyme and complement system) combined with the proteomic data (mimecan isoforms profile) could suggest modulation of the immune system or suppression of immune functions and may lead to viral infections in the animals. In fact, it is known that changes in the immune system may not represent a direct danger to the animals, but an organism weakened in its immune defenses is more susceptible to attacks by pathogens.

#### 3.2.5. ENA Assay

The ENA assay is a cytogenetic test that allows to evaluate the various alterations in the shapes of the nuclei of mature erythrocytes in addition to the micronucleus. In this ex vivo experiment, the ENA test shows clear patterns for all three treatments, with total anomalies values that in almost all cases are above the control value even if the statistical significance appears only for the treatment with MEHP (Figure 6). The most frequently encountered abnormalities are the lobed nuclei. The range of total anomalies values measured in this study are in line with those obtained by Casini et al. [12], where the nuclear abnormalities was evaluated in specimens of *C. caretta* from the Mediterranean. In that study, a correlation was reported between the presence of PAH and genotoxic effects in the blood as well as a correlation between comet assay and ENA assay. A paper by Cocci et al. [145] investigated the concentration of PAHs and PCBs in the blood of *C. caretta*; among the results obtained, a correlation was observed between the levels of PAHs and DNA methylation. These studies confirm the genotoxic potential of PAHs also for *C. caretta*. There are no exposure studies with PAHs and in particular chrysene using cells or tissues of this species; thus, the genotoxic potential of this compound is well-known in other animal classes [146,147,148,149].

To our knowledge, no studies on the potential genotoxic effects of MEHP or other phthalates on *C. caretta* were carried out, while information regarding other classes and species confirms the genotoxic potential of this metabolite of DEHP. Several authors [150,151] have shown that in humans MEHP is able to induce oxidation of DNA and single-strand breaks in DNA. Chang et al. [152] showed that this DEHP metabolite can determine DNA single breaks; moreover, MEHP determines the production of ROS, which could cause DNA damage. Iyama and Wilson [153] showed that MEHP causes both DNA oxidation damage and nucleotide damage in humans.

PBDEs have never been tested for genotoxic effects in *C. caretta* although some researchers found high PBDE concentrations in different sea turtles’ species [10,154,155,156]. If we explore the results obtained in other species, we see that in the work of He et al. [157], nervous system cells from mice were exposed to different concentrations (0, 2.06, 20.6, and 41.2 μM) of PBDE-47, and several biomarkers were subsequently evaluated, including damage to the DNA through the comet assay. As for the comet assay, the percentage of DNA damage was found to increase significantly at all concentrations tested compared to the control. Longo et al. [158] exposed human macrophage cell cultures to various concentrations (3, 6, 12, and 25 μM) of PBDE-47 for 24 and 48 h and evaluated the DNA fragmentation. At the highest concentration, the results showed a significant increase in DNA damage compared to the controls.

## 4. Conclusions

The main conclusions particularly focus on the novelties presented in this study. An ex vivo approach was developed and applied in *C. caretta* to evaluate the effects of direct contaminant exposure. This model mimics the in vivo system by preserving several of the properties that characterize and distinguish the system itself. In particular, the ex vivo model we proposed resembles the complexity of the investigated tissues by maintaining cellular and structural heterogeneity and several of the functional interconnections and correlations that the tissue components established in the living organism. Since alteration of the natural conditions is minimized in the ex vivo tissue cultures, the exposure responses we observed represent a precious compromise between in vitro and animal experiments.

Here, we tested the effects of three compounds of extreme interest for the toxicology and ecotoxicology of *C. caretta*: (i) the PAH chrysene; (ii) MEHP, a model compound for phthalates; (iii) and PBDE-47. These toxicants and relative classes are relevant in the marine natural environment, where they can also act synergistically.

We also proposed a 2D-PAGE/MS proteomic approach to investigate pollutant effects on the skin of loggerhead sea turtles. Our analyses highlighted specific contaminant signatures on the proteomic profile of the ex vivo skin slices, which reflect molecular and biochemical changes induced by each treatment. The overall synergistic evaluation of all the proteomic profiles we delineated by using PCA, correlation matrix, and spot-intensity heatmap stressed the relevance proteomics investigation may have in delineating specific biomarker modules for risk assessment of contaminant exposure. The differentially abundant protein spots we identified contributed to clarify the biochemical mechanisms underlying toxicity of chrysene, MEHP, and PBDE-47 in *C. caretta*. Notably, only GST-P was previously known and applied as a biomarker in ecotoxicology. Mimecan, PPIA, and S100A6 have not been previously described as directly affected by the pollutants we tested. Because of their several roles in tissue physiology, these proteins may represent novel potential biomarkers.

Genotoxic and immune system effects have been shown in the treated whole blood, highlighting the toxicological potential of the tested compounds for this species.

Proteomics and biomarker results confirm the suitability of the ex vivo system and indicate the potential risk the loggerhead sea turtle is undergoing in the natural environment.

It is worth to underline the potential of our ex vivo approach that might be applied to test the toxicity of other compounds and mixtures as well as to identify new biomarkers for predicting contaminant effects on *C. caretta* tissues. This would be of high relevance for a more holistic overview on the systemic effects of the tested contaminants.

## Figures and Tables

**Table 1:** Significant protein-spot differences from chrysene vs. MEPH; chrysene vs. PBDE-47; chrysene vs. DMSO; MEHP vs. PBDE-47; MEPH vs. DMSO; and PBDE-47 vs. DMSO comparisons, identified by mass spectrometry.

Spot N.	Protein Identification						Chrysene %V Mean ± SD	MEHP %V Mean ± SD	PBDE-47 %V Mean ± SD	DMSO CTRL %V Mean ± SD
	Protein ID	NCBIprot, UniProtKB, or GenBank AN	Score	Expected	Detected Masses/Matched Masses	Seq. Coverage %				
5	Actin. cytoplasmic 2 isoform X3 (*Cariama cristata*)	XP_009695579.1	92	0.0094	7/15	28	0.4141 ± 0.1171	0.1477 ± 0.1181	0 ± 0	0.1343 ± 0.1045
6	Alpha-enolase (*Chelonia mydas*)	XP_037737064.1	170	1.6 × 10^−10^	29/16	37	0 ± 0	0.3526 ± 0.09	0.1948 ± 0.0328	0.2042 ± 0.0774
8	Mimecan (*Chelonia mydas*)	EMP39737.1	75	0.05	4/4	15	0 ± 0	0.0478 ± 0.0063 ^€^	0.3156 ± 0.2172	0.5189 ± 0.3459 ^€^
10	Mimecan (*Chelonia mydas*)	XP_007055647.1	91	0.012	5/5	14	0 ± 0	0.7315 ± 0.1362 ^∑^	0.1901 ± 0.1724 ^∑^	0.3899 ± 0.1163
11	Mimecan (*Chelonia mydas*)	XP_007055647.1	108	0.00026	6/6	17	0.2121 ± 0.1567	0.3246 ± 0.097	0.1487 ± 0.7414 ^Ω^	0.4872 ± 0.1932 ^Ω^
12	Keratin. type I cytoskeletal 15 (*Chelonia mydas*)	XP_007062046.2	137	3.3 × 10^−7^	13/25	24	0.2075 ± 0.1123 *	0.7221 ± 0.1899 *	0.3926 ± 0.1333	0.5253 ± 0.2178
19	Glutathione S-transferase P (*Chelonia mydas*)	XP_037755179.1	148	2.6 × 10^−8^	11/17	47	0.00962 ± 0.0572 ^# ¥^	0.1725 ± 0.085	0.6684 ± 0.3708 ^¥^	0.2494 ± 0.1771 ^#^
22	Peptidyl-prolyl cis-trans isomerase A (*Chelonia mydas*)	XP_007066086.1	90	0.017	6/14	29	0.1531 ± 0.4331 *	0.8279 ± 0.1072 *	0.3072 ± 0.1801	0.4633 ± 0.2387
23	Hemoglobin subunit alpha-A (*Caretta caretta*)	Q10732 (Q10732.1)	132	1.00 × 10^−6^	8/11	57	0 ± 0	0.2615 ± 0.0819	0.1461 ± 0.0151	0.5494 ± 0.271
25	Protein S100-A6 (*Chelonia mydas*)	XP_037739342.1	LNDAEIVGLMEDLDR Score: 136; E: 5 × 10^−9^				0.08945 ± 0.0079 *	0.3056 ± 0.0909 *	0.1904 ± 0.014	0.155 ± 0.0105

* # ¥ € ∑ Ω Symbols indicate significant quantitative differences occurring between the four tested conditions: *, chrysene vs. MEPH; ¥, chrysene vs. PBDE-47; #, chrysene vs. DMSO; ∑, MEPH vs. PBDE-47; €, MEHP vs. DMSO; Ω, PBDE-47 vs. DMSO.

## Data Availability

Data sharing not applicable.

## Supplementary Materials

The following supporting information can be downloaded at: https://www.mdpi.com/article/10.3390/ijerph19074369/s1, Table S1. Significant protein spot differences from chrysene vs. MEPH; chrysene vs. PBDE-47; chrysene vs. DMSO; MEHP vs. PBDE-47; MEPH vs. DMSO; and PBDE-47 vs. DMSO comparisons.

## Abbreviations

% Vol: percentage relative volume; 2D: Two-Dimensional; 2-DE: Two-Dimensional Electrophoresis; 2D-PAGE: Two-Dimensional Polyacrylamide Gel Electrophoresis; ABTS: 2,2-Azino-di (3-thylbenzthiazoline); ACN: acetonitrile; BDE-47: 2,2′,4,4′-Tetrabromodiphenyl; CHCA: α-cyano-4-hydroxycinnamic acid; ChHV5: chelonid herpesvirus 5; CITES: Convention on International Trade in Endangered Species of Wild Fauna and Flora; COX-2: cyclooxygenase-2; DEHP: di-2-ethylhexyl phthalate; DMSO: dimethyl sulfoxide; DNA: deoxyribonucleic acid; EDC: endocrine-disrupting chemical; ENA: erythrocytic nuclear abnormalities; FP: fibropapillomatosis; GST-P: glutathione S transferase P; HEL: chicken egg-white lysozyme; HPA: hypothalamic–pituitary–adrenal; IEF: isoelectric focusing; IUCN: International Union for Conservation of Nature; MALDI: Matrix-Assisted Laser Desorption/Ionization; MEHP: mono-2-ethylhexyl phthalate; MS: mass spectrometry; MW: molecular weight; NBT: Nitroblue Tetrazolium; OOCs: organochlorine contaminants; OGN: mimecan/osteoglycin; PAH: polycyclic aromatic hydrocarbon; PBDE-47: polybrominated diphenyl ether-47; PC: principal components; PCA: Principal Component Analysis; PGE2: prostaglandin E2; pI: isoelectric point; PPIA: peptidyl-prolyl cis-trans isomerase A; ROS: reactive oxygen species; RPMI: Roswell Park Memorial Institute; S100A6: calcyclin; TAS: total antioxidant status; TFA: trifluoroacetic acid; TL: TissueLyser II; TOF: time of fly; VEGF: vascular endothelial growth factor.
