# Supplementary material for: A Novel Ex Vivo Approach Based on Proteomics and Biomarkers to Evaluate the Effects of Chrysene, MEHP, and PBDE-47 on Loggerhead Sea Turtles (Caretta caretta)
Source: Int J Environ Res Public Health. 2022 Apr 5;19(7):4369. doi: 10.3390/ijerph19074369 (PMC8998652; doi:10.3390/ijerph19074369)
Supplement: Supplementary file 1 [file ijerph-19-04369-s001.zip › ijerph-1560461-supplementary.pdf]

Table S1. Significant protein-spot differences from chrysene *vs* MEPH; chrysene *vs* PBDE-47; chrysene *vs* DMSO; MEHP *vs* PBDE-47; MEPH *vs* DMSO; PBDE-47 *vs* DMSO comparisons.

Spot N.	Chrysene %V Mean ± SD	MEHP %V Mean ± SD	PBDE-47 %V Mean ± SD	DMSO CTRL %V Mean ± SD
1	0 ± 0	0.6009 ± 0.286	0.3919 ± 0.214	0.1779 ± 0.073
2	0 ± 0	0.3717 ± 0.262	0.5072 ± 0.2218	0.5331± 0.3427
3	0.1492 ± 0.0061$^{¥}$	0.2116 ± 0.077	0.4569 ± 0.3101$^{¥}$	0.3082 ± 0.1803
4	0 ± 0	0.6557 ± 0.3713	0.3928 ± 0.2346	0.3308 ± 0.2184
5	0.4141 ± 0.1171	0.1477 ± 0.1181	0 ± 0	0.1343 ± 0.1045
6	0 ± 0	0.3526 ± 0.09	0.1948 ± 0.0328	0.2042 ± 0.0774
7	0 ± 0	0.1649 ± 0.1383	0.2251 ± 0.1766	0.4913 ± 0.2373
8	0 ± 0	0.0478 ± 0.0063$^{€}$	0.3156 ± 0.2172	0.5189 ± 0.3459$^{€}$
9	0.3169 ± 0.2807	0.4774 ± 0.2557	0 ± 0	0.1829 ± 0.0433
10	0 ± 0	0.7315 ± 0.1362$^{\Sigma}$	0.1901 ± 0.1724$^{\Sigma}$	0.3899 ± 0.1163
11	0.2121 ± 0.1567	0.3246 ± 0.097	0.1487 ± 0.7414$^{\Omega}$	0.4872 ± 0.1932$^{\Omega}$
12	0.2075 ± 0.1123*	0.7221 ± 0.1899*	0.3926 ± 0.1333	0.5253 ± 0.2178
13	0 ± 0	03013 ± 0.2366	0.5129 ± 0.1488	0.1943 ± 0.1484
14	0.1309 ± 0.4523$^{¥}$	0.3323 ± 0.1257	0.5085 ± 0.3915$^{¥}$	0.2547 ± 0.1402
15	0 ± 0	0 ± 0$^{€}$	0.1768 ± 0.196	0.487 ± 0.2912$^{€}$
16	0 ± 0	0.0953 ± 0.0652	0.307 ± 0.3141$^{\Omega}$	0 ± 0$^{\Omega}$
17	0 ± 0	0.3576 ± 0.2764	0.4288 ± 0.3443	0.2038 ± 0.1041
18	0 ± 0	0.3252 ± 0.3543	0.7298 ± 0.2431	0.5789 ± 0.228
19	0.00962 ± 0.0572$^{\#¥}$	0.1725 ± 0.085	0.6684 ± 0.3708$^{¥}$	0.2494 ± 0.1771$^{\#}$
20	0 ± 0	0.3857 ± 0.1803	0.2194 ± 0.1084	0.2734 ± 0.2482

21	0.5292±0.2991	0.2499±0.2777	0±0	0±0
22	0.1531±0.4331*	0.8279±0.1072*	0.3072±0.1801	0.4633±0.2387
23	0±0	0.2615±0.0819	0.1461±0.0151	0.5494±0.271
24	0.408±0.0099	0.6274±0.2251	0.2892±0.1049Ω	0.3272±0.1131Ω
25	0.08945±0.0079*	0.3056±0.0909*	0.1904±0.014	0.155±0.0105
26	0±0	0.5312±0.3475Σ	0±0Σ	0.1699±0.0372

* #¥ € ΣΩ Symbols indicate significant quantitative differences occurring between the four tested conditions: * chrysene *vs* MEPH; ¥ chrysene *vs* PBDE-47; # chrysene *vs* DMSO; Σ MEPH *vs* PBDE-47; € MEHP *vs* DMSO; Ω PBDE-47 *vs* DMSO.
